# Open versus minimally invasive fixation of a simulated syndesmotic injury in a cadaver model

**DOI:** 10.1186/s13018-017-0658-0

**Published:** 2017-10-27

**Authors:** Adam C. Shaner, Norachart Sirisreetreerux, Babar Shafiq, Lynne C. Jones, Erik A. Hasenboehler

**Affiliations:** 1 Department of Orthopaedic Surgery, The Johns Hopkins University, 600 N Caroline Street, Baltimore, MD 21287 USA; 2 Department of Orthopaedics, Faculty of Medicine, Ramathibodi Hospital, Mahidol University, 270 Rama VI Rd, Ratchathewi, Bangkok, 10400 Thailand; 3 Department of Orthopaedic Surgery, The Johns Hopkins University/Johns Hopkins Bayview Medical Center, 4940 Eastern Ave., #A667, Baltimore, MD 21224-2780 USA

**Keywords:** Ankle, Fibula, Minimally invasive technique, Open technique, Syndesmotic injury

## Abstract

**Background:**

Malreduction of unstable syndesmotic ankle fractures is common. This study compared the reduction quality of an anterolateral open technique (OT) versus a conventional minimally invasive technique (MIT).

**Methods:**

Fourteen fresh-frozen lower torso specimens with 28 matched lower extremities underwent computed tomography (CT) to measure syndesmosis position before dissection. Reduction was performed using direct visualization and fluoroscopy for the OT group (right-sided specimens) and fluoroscopy only for the MIT group (left-sided specimens). Fixation was achieved with 2 cortical screws. Measurements were repeated with postfixation CT scans. Statistical analysis used a two-tailed *t* test (*α* = 0.05).

**Results:**

Mean posterior fibula-tibia distance decreased after OT by 0.3 ± 0.5 mm and increased after MIT by 0.7 ± 0.6 mm (*P* = 0.025 for difference between techniques). Mean anterior fibula-tibia distance decreased after OT by 0.4 ± 0.2 mm (*P* = 0.007) and did not change significantly after MIT (− 0.01 ± 0.4 mm (*P* = 0.686). Mean anterior translation after OT was 0.04 ± 0.4 mm (*P* = 0.856), and mean posterior translation after MIT was 0.3 ± 0.7 mm (*P* = 0.434). Mean medialization after OT was 0.3 ± 0.4 mm (*P* = 0.132), and mean lateralization after MIT was 0.2 ± 0.6 mm (*P* = 0.446).

**Conclusions:**

Both techniques produced near-anatomic reduction of the fibula, with MIT producing significantly more internal rotation malreduction than OT. OT appears to restore near-anatomic fibula position, although this did not differ significantly from the results of MIT. We conditionally recommend OT when closed reduction of the syndesmosis cannot be obtained.

## Background

Unstable rotational ankle fractures with associated syndesmotic disruption are common, with approximately 20% of operatively treated fractures requiring syndesmosis fixation [1]. Achieving anatomic syndesmosis reduction intraoperatively is important but challenging. The optimal method for treating these injuries is debated in the literature, in regard to proper implant selection (screws versus suture), positioning of the ankle during repair, and initiation of postoperative weightbearing. Further, the number of fixation cortices (3 versus 4) is also debated because instrumentation loosening or failure can occur, respectively. Hence, a “gold standard” of treatment for these injuries has yet to be described [2, 3]. Despite advanced imaging modalities and fixation techniques [4, 5], malreduction risks remain high, with a reported rate of > 50% [6–8]). Suboptimal clamp position during open or closed reduction can lead to malreduction and may not be apparent with standard intraoperative imaging [9–13].

Intraoperative 3D fluoroscopy can be used to assist with assessment of ankle reduction [4]. Franke et al. [14] assessed syndesmosis reduction quality intraoperatively, comparing 3D fluoroscopy with standard fluoroscopy in 2286 ankle fractures. This comparison resulted in a revision of the reduction in 33% of cases, improving it in 31% of cases [14]. However, advanced intraoperative imaging is expensive, not always available, and associated with a higher radiation dose compared with standard fluoroscopy. Furthermore, Davidovitch et al. [15] showed that intraoperative 3D fluoroscopy did not decrease the rate of syndesmosis malreduction in 36 patients.

Anatomic variation presents another challenge to reduction. Studies by Nault et al. [16] and Shah et al. [17] have shown significant anatomic variation of the distal tibiofibular joint, which has been described for both standard fluoroscopic measurements, as well as axial computed tomography (CT) imaging [18]. Mukhopadhyay et al. [19] showed that when comparing the injured ankle with the contralateral (uninjured) ankle fluoroscopically, syndesmosis diastasis can be improved significantly by using fluoroscopy, as opposed to standard reduction methods alone. These studies emphasize the importance of evaluating the anatomic position of the uninjured ankle and using an individualized approach to intraoperative reduction assessment.

Recent studies evaluating open reduction of the syndesmosis have shown improved reduction quality [20, 21], potentially reducing the need for advanced intraoperative imaging. The purpose of this study was to compare the reduction quality of two reduction techniques, an anterolateral open technique (OT) versus a conventional minimally invasive technique (MIT) for a syndesmotic injury. Using a simulated syndesmotic cadaveric model, we measured the width and reduction of the syndesmotic joint with preinjury and postreduction CT scans. We hypothesized that an anterolateral open approach with direct visualization of the syndesmosis would result in a lower rate of malreduction compared with a standard closed reduction and clamping technique using 2D fluoroscopy.

## Methods

**Fig. 1:**
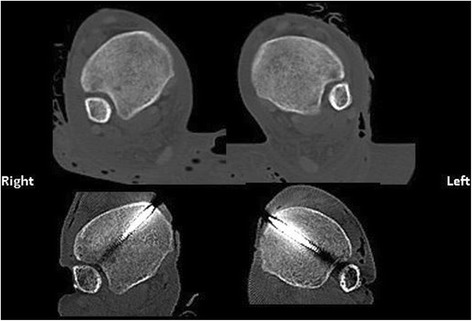
Example of computed tomography scan axial images before and after reduction with open technique (right side) and minimally invasive technique (left side). Reduction appears to be almost identical to the predissection condition for both techniques

Fourteen fresh-frozen lower torso specimens (3 females) with 28 matched lower extremities were obtained from the Maryland State Anatomy Board. Dual-energy X-ray absorptiometry scans were obtained for all specimens to ensure adequate bone quality. Specimens had a mean *T* score of − 0.39 (range, − 2.2 to 2.3) and a mean Z score of 0.71 (range, − 0.9 to 3.5) [22]. The mean age at the time of death was 77 ± 13 years. None of the specimens had a history of surgery in either ankle. All specimens underwent bilateral lower extremity CT scans with 1.5 mm cuts to measure the anatomic syndesmosis position (Fig. 1). The picture archiving and communication system software used for all CT scans (Emageon, Inc., UltraVisual Medical Systems, Birmingham, AL) allowed us to obtain precise measurements up to the fifth decimal place.

**Fig. 2:**
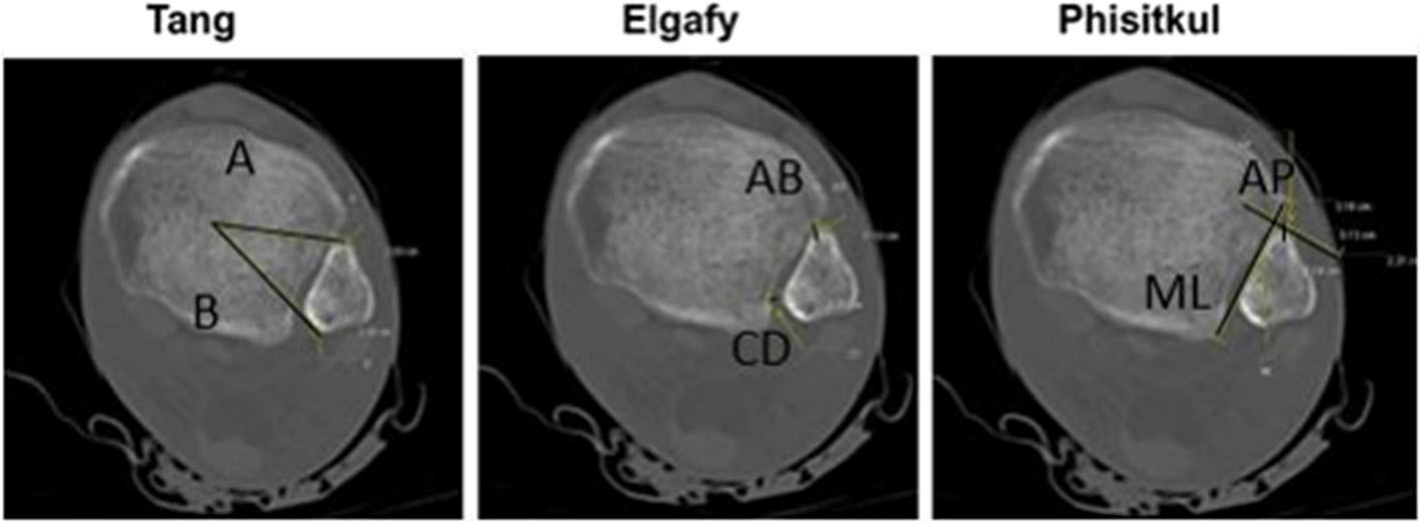
UltraVisual imaging software (Emageon, Inc., UltraVisual Medical Systems, Birmingham, AL) was used to perform measurements on the axial computed tomography scan view at 1 cm above the distal tibia articular surface. Tang et al. [23], ratio of A:B distance reflects fibular rotation. Elgafy et al. [24] and Phisitkul et al. [25] methods, direct measurements of anterior (AB and AP) and posterior (CD and ML) tibial-fibular distance, represent fibular translation in the coronal and sagittal planes, respectively

Measurements of the distal tibiofibular joint were obtained from the axial view at 1 cm from the distal tibial articular surface and were performed according to the techniques described below (Fig. 2).

### Method of Tang et al. [23]

The center of the distal tibial metaphysis was established, and the distance (in cm) from this point was measured to the anterior (A) and posterior (B) fibular cortices. The ratio of A:B reflects the relative rotation of the fibula with values of < 1.0 cm indicating relative internal rotation and values > 1.0 cm representing relative external rotation.

### Method of Elgafy et al. [24]

The distance (in mm) was measured between the closest point on the anterior tubercle of the tibia and the point on the fibula closest to that location (AB). A second measurement (in mm) was obtained between the point on the fibula that is midway between the medial-most and the posterior-most points, and the location on the tibia that is closest to that location (CD). Both the mean of the anterior and posterior measurements, as well as the difference between the anterior and posterior values (AB – CD) were calculated. A positive value indicates greater anterior distance (relative external rotation). A negative value indicates greater posterior distance (relative internal rotation).

### Method of Phisitkul et al. [25]

The medial-lateral distance was measured (in mm) from the medial-most border of the fibula to a line connecting the anterior and posterior tubercles of the tibia. The anterior-posterior distance was measured between a line perpendicular to the tubercular line at the anterior tubercle and the anterior-most point of the fibula. Positive numbers denote posterior translation, and negative numbers denote anterior translation. For medial-lateral translation, positive numbers denote medial translation, and negative numbers denote lateral translation.

### Injury simulation

**Fig. 3:**
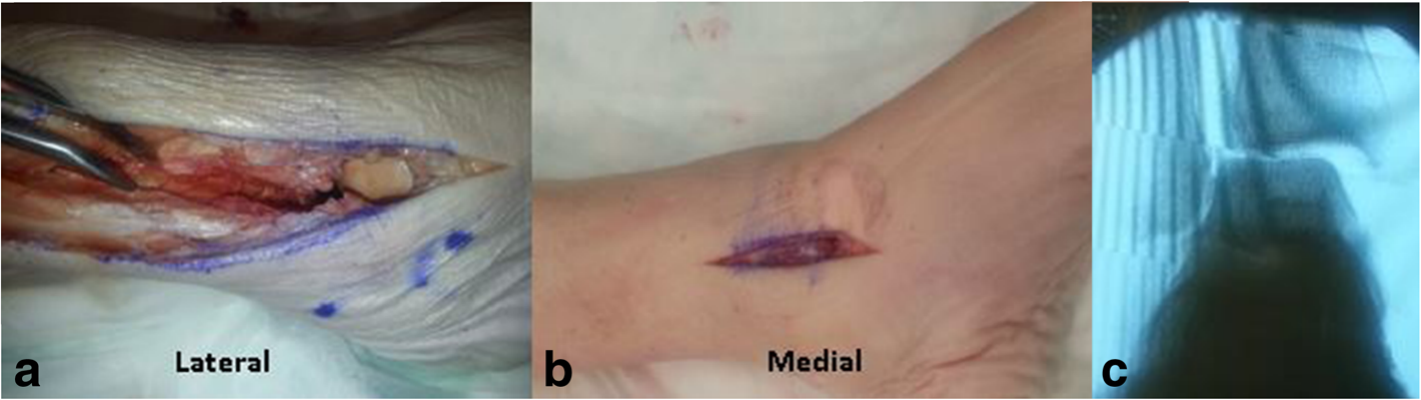
**a** Anterolateral skin incision over the distal fibula and syndesmosis carried out more proximally to allow dissection of the interosseous membrane. The lamina spreader was used to visualize the PITFL and transect it. **b** Medial skin incision was made to transect the deltoid ligament. **c** External rotation stress view confirming instability

Specimens underwent dissection and syndesmosis ligamentous division to simulate an unstable ankle injury using a previously described method [26]. All right-sided lower extremities underwent open dissection with an anterolateral approach to the anterior syndesmosis (Fig. 3a). The anterior inferior tibiofibular ligament (AITFL) was visualized directly and divided, along with the interosseous ligament and the distal 3 cm of the interosseous membrane. The fibula was then translated laterally using a lamina spreader, and the posterior inferior tibiofibular ligament (PITFL) was visualized and fully transected through the syndesmosis. The deep deltoid ligaments were then divided completely through a second longitudinal medial malleolus incision (Fig. 3b). Instability of the ankle with syndesmosis widening was confirmed using an external rotation stress test under direct and fluoroscopic visualization (Fig. 3c).

**Fig. 4:**
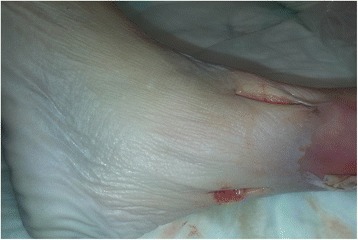
For the minimally invasive technique, two incisions were made (anterior and posterior) over the AITFL and PITFL. Similar to Fig. 3, a medial skin incision was made over the medial malleolus to transect the deltoid ligament. A stress view was taken after preparation and under fluoroscopy, as shown in Fig. 3

All left-sided lower extremities underwent ligamentous division using minimally invasive techniques with separate incisions anterior and posterior to the syndesmosis to divide the AITFL, PITFL, and interosseous membrane (Fig 4a). A medial malleolus incision was used to divide the deep deltoid ligaments (Fig. 3b, similarly as for OT). An external rotation stress test using fluoroscopy was performed to verify instability (Fig. 3c). A large Weber clamp was used to obtain reduction in both techniques.

### Syndesmosis reduction

#### Open approach

**Fig. 5:**
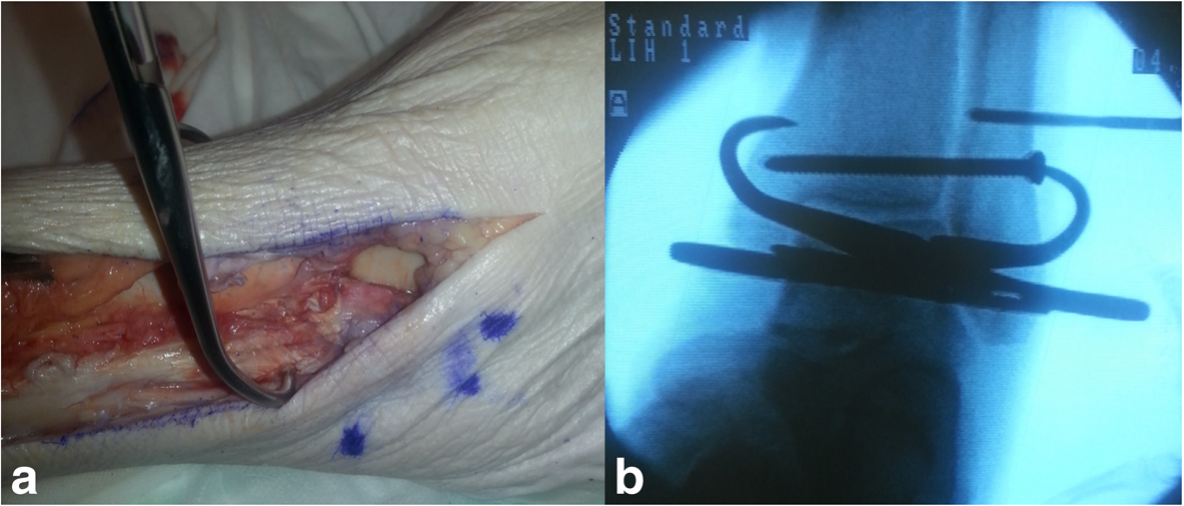
**a** Clamp position with direct visualization of syndesmosis reduction anteriorly. **b** Fluoroscopic verification of clamp position, reduction of syndesmosis on mortise view, and placement of two 3.5-mm quadricortical screws

For the open approach, the syndesmosis was reduced under direct visualization, ensuring reduction of the fibular articulation with the tibia and position at the anterior incisura. Weber clamp tines were placed approximately 1 cm above the plafond, on the lateral malleolar ridge of the fibula and over the center of the anteroposterior width of the tibia medially. This allowed coaxial compression in the plane of the syndesmosis. Clamp position was verified and adjusted as needed using fluoroscopy (Figs. 1 and 5a, b). The clamp was adjusted to 4–5 clicks to standardize the amount of compression while holding the foot in neutral dorsiflexion. Subsequently, quadricortical trans-syndesmosis fixation was placed in classic fashion using 3.5-mm cortical screws (DePuy Synthes, Paoli, PA) from lateral to medial, after predrilling with a 2.5-mm drill bit. Quadricortical fixation is the authors’ preferred method because this technique can provide better syndesmotic stability versus tricortical fixation, which can lead to instrumentation loosening. Two parallel 3.5-mm cortical screws were placed approximately 0.5 and 1.5 cm above and parallel to the tibial plafond and approximately 30° from posterior to anterior in the horizontal plane. Proper position was verified using fluoroscopy (Fig. 5b).

#### Minimally invasive approach

For the minimally invasive approach, a 3-cm lateral incision was made over the distal fibula at the level of the tibiofibular joint. To obtain coaxial compression at the level of the syndesmosis, we placed Weber clamp tines similarly to the open approach (1 cm above the plafond on the lateral malleolar ridge of the fibula and over the center of the anteroposterior width of the tibia medially). Clamp position was verified and adjusted as needed using fluoroscopy, and reduction was judged using standard anteroposterior/mortise and perfect lateral fluoroscopic views. The clamp was adjusted to 4–5 clicks to standardize the amount of compression. Syndesmosis fixation was performed in a similar fashion to the OT, through a small lateral incision (Figs. 1 and 5a, b).

Postfixation CT scans were performed for all ankles, and measurements were obtained again using all three methods. Pre- and postdissection measurements were compared to evaluate reduction.

### Statistical analysis

The data were collected and analyzed using an electronic spreadsheet (Excel 2007, Microsoft, Redmond, WA). Mean values and differences with 95% confidence intervals were calculated. To compare anatomic differences with regard to laterality on the same cadaver, we used an unpaired two-tailed *t* test for the predissection measurements of the left versus right side. The position of the fibula within the incisura before and after fixation was compared between the OT and MIT groups for all specimens using an unpaired two-tailed *t* test. A paired two-tailed *t* test was used to compare differences between pre- and postfixation measurements for the same specimen. *P* values less than 0.05 were considered statistically significant.

## Results

No significant differences in preinjury fibular anatomic position were found between right and left lower extremities from the same cadaver (Table 1).

**Table 1:** Computed tomography-based measurements of 14 matched-pair cadaveric ankles before dissection and after fixation of a simulated syndesmotic injury using OT versus MIT

Measure	Predissection measurements (mean ± SD), mm		*P* value	Postfixation difference (mean ± SD), mm				*P* value for OT versus MIT
	Right ankles	Left ankles		OT	*P* value	MIT	*P* value	
Tang et al. [23]								
A^a^	29 ± 1.1	28 ± 1.1	0.338					
B^b^	30 ± 1.4	30 ± 1.4	0.749					
A:B^c^	0.97 ± 0.03	0.95 ± 0.03	0.412					
Change in A				− 0.2 ± 0.7	0.54	− 0.2 ± 0.5	0.50	0.937
Change in B				0.4 ± 1.1	0.45	0.1 ± 0.9	0.81	0.644
Change in A:B				− 0.02 ± 0.03	0.17	− 0.01 ± 0.02	0.39	0.485
Elgafy et al. [24]								
AB^d^	1.7 ± 0.3	1.4 ± 0.3	0.266					
CD^e^	3.0 ± 0.5	2.9 ± 0.5	0.871					
AB – CD^f^	− 1.2 ± 0.6	− 1.4 ± 0.4	0.614					
Change in AB				− 0.4 ± 0.2	0.007	− 0.01 ± 0.4	0.686	0.071
Change in CD				− 0.3 ± 0.5	0.338	0.7 ± 0.6	0.044	0.025
Change in AB − CD				0.1 ± 0.6	0.709	0.6 ± 0.8	0.133	0.288
Phisitkul et al. [25]								
Anteroposterior^g^	2.0 ± 0.3	2.1 ± 0.4	0.634	− 0.04 ± 0.4	0.856	0.3 ± 0.7	0.434	0.434
Mediolateral^h^	2.2 ± 0.5	2.2 ± 0.4	0.966	− 0.3 ± 0.4	0.132	0.2 ± 0.6	0.446	0.151

*MIT* minimally invasive technique, *OT* open technique, *SD* standard deviation

^a^“A” represents distance from center of distal tibial metaphysis to anterior fibular cortices

^b^“B” represents distance from center of distal tibial metaphysis to posterior fibular cortices

^c^The ratio of A:B reflects the relative rotation of the fibula

^d^“AB” represents distance between closest point on anterior tubercle of tibia and point on fibula closest to that location

^e^“CD” represents distance between point on fibula midway between medial-most and posterior-most points, and location on tibia closest to that location

^f^“AB – CD” represents difference between mean anterior and posterior values

^g^Medial-lateral distance from medial-most border of fibula to a line connecting anterior and posterior tubercles of the tibia

^h^Anterior-posterior distance between a line perpendicular to the tubercular line at the anterior tubercle and the anterior-most point of the fibula

Using the measurement technique of Tang et al. [23], and comparing postdissection versus predissection values for the same limb, we found that MIT produced a mean decrease in the distance from the tibial center to the anterior fibular cortex of 0.2 ± 0.5 mm, and OT produced a mean decrease of 0.2 ± 0.7 mm. The posterior distance increased by a mean of 0.1 ± 0.9 mm using MIT compared with 0.4 ± 1.1 mm using OT. Neither difference was statistically significant when compared with preinjury anatomic position, nor was the difference between techniques significant. Both MIT and OT resulted in relative decreases in the ratio of anterior to posterior distance of 0.01 ± 0.02 mm and 0.02 ± 0.03 mm, respectively. This indicates net internal rotation of the fibula with both methods (Table 1). The difference between methods was not statistically significant.

Using the method of Elgafy et al. [24], we observed a narrowing of the anterior syndesmosis of 0.4 ± 0.2 mm (*P* = 0.007) with OT. This was a significant difference compared with preinjury measurements but was not significantly different between techniques (*P* = 0.071). Conversely, MIT resulted in shortening of the anterior syndesmosis space by 0.01 ± 0.4 mm, but this was not statistically significant (*P* = 0.686). Measurement of the posterior syndesmosis distance showed a decrease of 0.3 ± 0.5 mm with OT, which was not a significant change (*P* = 0.338). MIT showed an increase in this distance of 0.7 ± 0.6 mm. This was significant not only compared with the prefixation anatomic fibular position (*P* = 0.044) but also compared with OT (*P* = 0.025). When analyzing the change in overall distance using the difference between the anterior and posterior measurements, neither OT (0.1 ± 0.6 mm, *P* = 0.709) nor MIT (0.6 ± 0.8 mm, *P* = 0.133) significantly changed the overall position of the fibula, with a comparative *P* value of 0.288 (Table 1).

Lastly, using the measurement method of Phisitkul et al. [25], we found that OT produced 0.04 ± 0.4 mm of anterior translation compared with preinjury position (*P* = 0.856), whereas MIT produced posterior translation of 0.3 ± 0.7 mm (*P* = 0.434), with no difference between groups (*P* = 0.434). Measurement of medial-lateral translation resulted in a net 0.3 ± 0.4 mm (*P* = 0.132) of medialization using OT and a relative lateralization of 0.2 ± 0.6 mm (*P* = 0.446) with MIT. Again, there was no significant difference between groups (*P* = 0.151).

## Discussion

Syndesmotic ankle injuries are challenging to treat, with patients having pain and radiographic widening at 5-year follow-up in as many as 60% of cases [27]. Malreduction of the syndesmosis has been consistently shown to result in poor long-term outcomes, with ankle stiffness and poor functional outcome scores [1, 7].

With both techniques, we were able to restore the syndesmosis to near-anatomic (predissection) position. The quality of reduction was acceptable in the MIT and OT groups, with no malreductions > 0.2 mm using any of the measurement techniques. However, even with direct visualization of the AITFL and anterior incisura and proper clamp positioning using appropriate visualization and fluoroscopy, there was a propensity for a decrease in the anterior fibula-incisura distance of up to 0.4 mm. This was the only significant difference we found with OT, and it did not occur with increased anterior-posterior translation or posterior fibular rotation. This suggests that there was a net compression effect or medial translation, rather than rotational malreduction. This finding is consistent with studies by Haynes et al. [28] and Cherney et al. [29] that showed overcompression was likely during reduction clamping of the syndesmosis, with a mean of 1 mm of overcompression and 5° of external rotation. Similar results were found in a cadaveric study by Phisitkul et al. [25], which showed a mean syndesmosis displacement of 0.1 ± 0.77 mm in all degrees of instability and overcompression of 0.93 ± 0.70 mm during clamping, with the clamp in the neutral anatomical axis.

Use of intraoperative imaging to assess reduction quality is challenging. A cadaver study by Marmor et al. [10] showed that as much as 30° of external rotation may be undetectable using intraoperative fluoroscopy. Franke et al. [14] used intraoperative 3D fluoroscopy scanning to assess reduction performed under fluoroscopy and found malreduction in 33% (82 of 251) of cases. Even with the use of intraoperative 3D scanning, Davidovitch et al. [15] showed a 31% (5 of 16) malreduction rate, compared with a 25% (5 of 20) malreduction rate with standard fluoroscopic imaging [30].

Alternative fixation methods such as a suture endobutton have been described. Although this technique may improve reduction by allowing physiologic motion at the syndesmosis, this method has not been shown to prevent syndesmosis malreduction completely [30]. Further, other than avoiding removal of instrumentation after 1 year or secondary to instrumentation failure or loosening as seen with screw fixation, clinical and radiographic outcomes at 1 year have not shown statistically significant differences between endobutton and screw fixation [31, 32].

Recently, open reduction and debridement of the syndesmosis has been shown to result in improved reduction rates. However, a greater amount soft tissue dissection is necessary for this approach [7, 21].

Miller et al. [20] reported a decreased rate of malreduction using direct visualization of the PITFL and posterior malleolus. A 16% malreduction rate (24 of 149 ankles) was found in ankles in which the posterior syndesmosis and posterior malleolus were fixed with direct visualization, compared with a 52% (13 of 24) malreduction rate in ankles fixed with indirect and fluoroscopic reduction only. In our cadaver model, the PITFL was divided completely but not repaired because no posterior malleolus fragment was present. This may have contributed to the similar results for the OT and MIT groups in our study.

Open reduction of the anterior syndesmosis was superior to the MIT in preventing overall internal rotation malreduction of the fibula based on the increase in posterior fibula-incisura distance. The MIT produced an increase in this distance of 0.7 ± 0.6 mm, which was significant not only compared with the anatomic (prefixation) fibular position (*P* = 0.044) but also compared with OT (*P* = 0.025). With no associated net change in the anterior fibula-incisura distance, this represents a purely internal rotation malreduction of the fibula, similar to that found by Davidovitch et al. [15].

This type of malreduction likely results from improper clamp positioning during reduction [11, 25, 33]. Clamp overcompression could also be a reason for malreduction, and using a calibrated clamping device during reduction, with or without the aid of advanced intraoperative imaging, might be necessary. We attempted to standardize this by limiting compression to 4–5 clicks with the clamp. However, the clinical relevance of this possible overtensioning has yet to be determined.

Our study has several strengths. We were able to reliably simulate a syndesmotic injury with a reproducible amount of instability using a cadaver model. Furthermore, pre- and postdissection CT scans allowed for accurate assessment of patient anatomy for determining quality of reduction after fixation. The precision of the measurements obtained through the software we used (Emageon UltraVisual) might not be clinically relevant; however, we believe that such precision increases the reliability of our reduction methods. We controlled for anatomic variability by using matched-pair cadaver ankles.

This study also has several limitations. Dissection and fixation were performed in cadaveric ankles, in which bone density and the quality of skin, tendon, and articular tissues differ from the in vivo state. This may influence measurements and affect the validity of our model; however, bone density was verified and controlled for with pre-evaluation dual-energy X-ray absorptiometry scanning. The type of reduction clamp (e.g., large Weber, periarticular, or collinear) is a surgeon-specific choice. We used a larger, pointed Weber clamp because of the thin habitus of our specimens. Another weakness of our study is that our model reflected a purely ligamentous injury of the ankle without associated high fibular fracture as typically seen in the clinical setting. Syndesmotic injuries with associated fibular fractures can be difficult to reduce because any malreduction of the fibula increases the likelihood of syndesmosis malreduction. That said, anatomic fibula reduction is usually achieved for distal fibula shaft fractures, whereas more proximal fractures are left unreduced because displacement is minimal and considered to be clinically unimportant. On the basis of this consideration, we chose a purely ligamentous injury model, as used in previous ankle studies. This model was easily reproducible, thereby eliminating the variability of fracture size, location, and fixation options. The absence of a repaired PITFL in our model may also have affected overall reduction because anatomic reduction of the posterior malleolus or direct reduction of PITFL injuries has been shown to restore rotational fibular stability similar to syndesmosis fixation [21, 34].

## Conclusion

Our study indicates that the quality of syndesmosis reduction achieved using MIT (with conventional 2D fluoroscopy) is comparable to that achieved using OT (with direct visualization). MIT produced more internal rotation malreduction of the fibula compared with OT. However, there was no significant difference in fibula reduction between the two techniques. Therefore, we can only conditionally recommend OT for the reduction of the syndesmosis. This technique might be useful when reduction of the syndesmosis cannot be obtained using MIT (e.g., in cases of interposition of soft tissue). Randomized clinical trials are needed to validate these findings and to provide further insight into optimal treatment of these challenging injuries.

## Abbreviations

AITFL: Anterior inferior tibiofibular ligament
CT: Computed tomography
MIT: Minimally invasive technique
OT: Open technique
PITFL: Posterior inferior tibiofibular ligament
